# Current status and impending progress for cassava structural genomics

**DOI:** 10.1007/s11103-020-01104-w

**Published:** 2021-02-18

**Authors:** Jessica B. Lyons, Jessen V. Bredeson, Ben N. Mansfeld, Guillaume Jean Bauchet, Jeffrey Berry, Adam Boyher, Lukas A. Mueller, Daniel S. Rokhsar, Rebecca S. Bart

**Affiliations:** 1 Department of Molecular & Cell Biology, University of California, Berkeley, CA 94720 USA; 2 Innovative Genomics Institute, University of California, Berkeley, CA 94720 USA; 3 Donald Danforth Plant Science Center (DDPSC), St. Louis, MO 63132 USA; 4 Boyce Thompson Institute, Ithaca, NY 14853 USA; 5 DOE Joint Genome Institute, Walnut Creek, CA USA; 6 Chan-Zuckerberg BioHub, 499 Illinois, San Francisco, CA 94158 USA

**Keywords:** Cassava, Genomics, Crop improvement, Phased genomes, Heterozygous genomes

## Abstract

**Key message:**

We demystify recent advances in genome assemblies for the heterozygous staple crop cassava (*Manihot esculenta*), and highlight key cassava genomic resources.

**Abstract:**

Cassava, *Manihot esculenta* Crantz, is a crop of societal and agricultural importance in tropical regions around the world. Genomics provides a platform for accelerated improvement of cassava’s nutritional and agronomic traits, as well as for illuminating aspects of cassava’s history including its path towards domestication. The highly heterozygous nature of the cassava genome is widely recognized. However, the full extent and context of this heterozygosity has been difficult to reveal because of technological limitations within genome sequencing. Only recently, with several new long-read sequencing technologies coming online, has the genomics community been able to tackle some similarly difficult genomes. In light of these recent advances, we provide this review to document the current status of the cassava genome and genomic resources and provide a perspective on what to look forward to in the coming years.

## Introduction

*Manihot esculenta* Crantz, referred to as cassava, yuca, or manioc, among other common names, is an important staple food source for over a billion people in tropical regions around the globe (Lebot, 2019). This crop develops large storage roots that are harvested for starch. Cassava leaves are also eaten but are considered a relatively minor source of calories. Cultivated cassava (*M. esculenta* ssp. *esculenta*) was domesticated in the Amazon, likely from *M. esculenta* ssp. *flabellifolia* (Pohl) (Olsen 2004; Léotard et al. 2009; Olsen and Schaal 1999; Olsen and Schaal 2001; Hillocks et al. 2002). While *M. glaziovii* Muell. Arg., the Ceará or India rubber tree, is sometimes erroneously referred to as “wild cassava,” genome sequencing has confirmed that it is a distinct species that diverged from cassava approximately 3 million years ago (Bredeson et al. 2016). However, some cassava accessions contain genomic regions derived from *M. glaziovii*, the result of an interbreeding program in the 1930s intended to confer disease resistance (Jennings 1976; Bredeson et al. 2016).

**Fig. 1:**
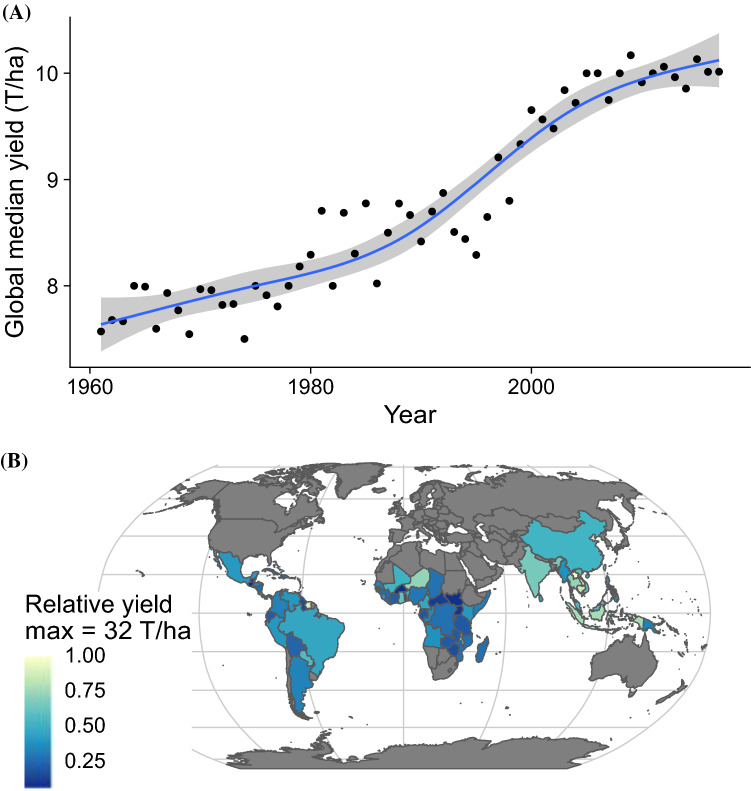
Global cassava yields show potential for improvement. **a** Cassava global median yields trends (1961–2017). Natural cubic spline smoothed trendline (blue) and standard errors (shaded ribbon). Cassava yields rose significantly since the 1980s, possibly due to improvements in germplasm and breeding. More recently however, there has been a plateau in yields. **b** Large disparity in global yields around cassava producing regions also suggests there is still potential for large scale gains. Yields in each cassava producing country plotted relative to the maximum produced in 2017 (32 Tons/ha, Lao People’s Democratic Republic) (Source: FAOSTAT, December 2019)

In contrast to the majority of staple food sources that are planted each year from botanical seed, such as cereals and legumes, cassava is propagated clonally through stem cuttings. A relevant consequence of this agricultural practice is that it circumvents sexual reproduction. Among sexual organisms, recombination that occurs during meiosis is a major driver of diversification that fuels evolution. Thus, in clonally propagated plants like cassava, we might expect limited genetic diversity and a slower rate of evolution that ultimately yields a weak crop. However, there are thousands of distinct varieties of cassava and this crop is prized for agriculturally important phenotypes including tolerance to abiotic and biotic stresses and an ability to produce high yields without external inputs such as irrigation and chemical fertilizer (Food and Organization 2013). Cassava breeding is difficult, in part because of high levels of heterozygosity, asynchronous flowering, and low seed set (Halsey et al. 2008). However, cassava is able to outcross and so through determined breeding efforts, significant increases in cassava yields have been observed over the mid and late 1900s (Ceballos et al. 2020); Fig. 1).

For an in depth review of cassava agricultural attributes, we point readers to existing, recent reviews (Food and Organization 2013; Fanou et al. 2018; Bart and Taylor 2017; Lin et al. 2019). In this review, we focus on recent advances within the cassava genomics community, highlighting publicly available resources and specific aspects of cassava that make genomics research on this crop difficult but fascinating. We compare and contrast different sequencing and genomics technologies and refer to examples from other organisms as a guide for future efforts on cassava. This review is targeted to a broad audience interested in generating and using genomics data for cassava research. Further, we hope this review will be useful and interesting to researchers outside the cassava community who are interested in heterozygous genomes.

## Cassava Reference Genome

The year 2019 marked the ten-year anniversary of the first public release of a cassava reference genome assembly, making this a fitting time to review the history of its development as a public resource. Cassava was one of the first “orphan” crop genomes to be sequenced, and the quality of the cassava genome sequence has steadily improved over the past decade, taking advantage of ongoing developments in genome sequencing and bioinformatics (Table 1). While plant chromosomes are tens to hundreds of megabases (Mb) in length, genome sequencers are only able to “read” shorter sequences (hundreds to thousands of base pairs, depending on technology). These reads are computationally combined, or “assembled,” in order to reconstruct chromosomal sequences. Here we briefly summarize the development of the cassava genome.

**Table 1:** Cassava AM560-2 reference genome assemblies

	v4.1	v5.1	v6.1	v7.1
Release	2009	2014	2016	2019
Primary sequence technology	454	454	Illumina	PacBio
Primary scaffolding data	454 mate pair	Composite genetic map	Illumina mate pair, fosmid, genetic maps	Illumina mate pair, fosmid, genetic maps
Total scaffold length	533 Mb	534 Mb	582 Mb	669 Mb
Total contig length	419 Mb	419 Mb	496 Mb	667 Mb
Number of chromosomal scaffolds	0	18	18	18
Bases in chromosomes	0	305 Mb	444 Mb	606 Mb
Contig N50 length (contiguity)	11 kb	11 kb	27 kb	693 kb
Annotated genes	30,666	30,666	33,033	33,849

**Fig. 2:**
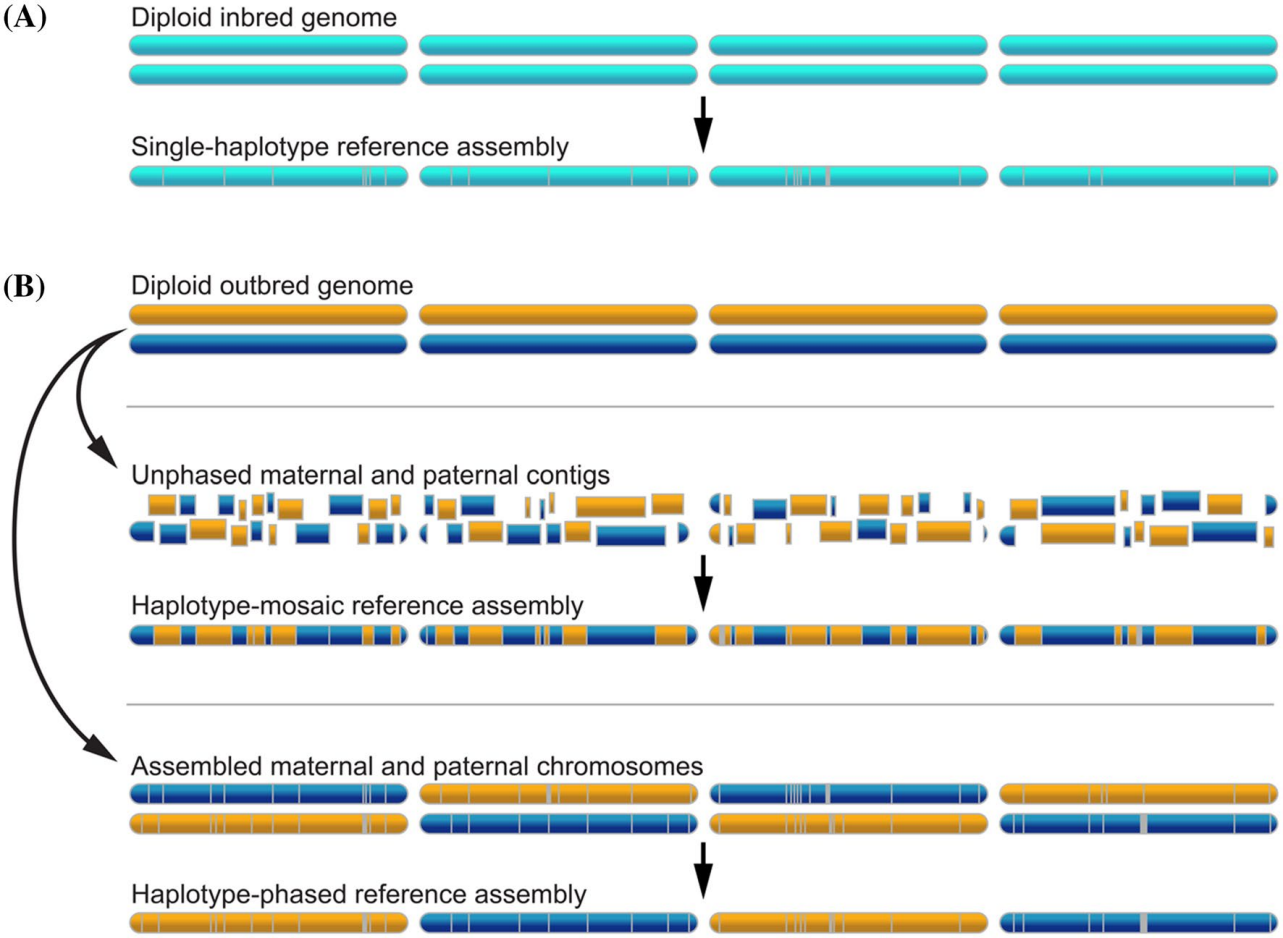
Reference genome assembly strategies. **a** Generating a haploid representation (reference assembly) of a diploid inbred genome is relatively straightforward. Due to homozygosity, sequence reads from the two haplotypes assemble together. **b** The heterozygosity present in a diploid outbred genome means that sequences from maternal and paternal haplotypes (blue and gold) will tend to assemble separately. In this case, to generate a haploid reference assembly, researchers can either combine maternal and paternal contigs into a haploid representation for each chromosome (haplotype-mosaic reference assembly), or they can try to fully assemble the maternal and paternal chromosomes, choosing one or the other to represent each chromosome in the reference (haplotype-phased reference assembly). Gray, assembly gaps

Cassava genomics has emphasized the “whole genome shotgun” approach, in which a large number of random, overlapping genome fragments (“reads”) are sequenced. Since many reads are redundantly derived from each genomic locus, such reads can be identified bioinformatically and combined to reconstruct the underlying genome sequence. For outbred species, however, a single individual has two haplotypes, inherited from its maternal and paternal parents (Fig. 2b). In *M. esculenta*, haplotypes typically differ by ~0.7% at the single nucleotide level, which may include short insertions and deletions. More dramatic differences (e.g., large insertions and structural rearrangements) may also be present but have not yet been comprehensively investigated. Thus, the task of assembly is greatly simplified if a homozygous genotype can be used as the reference, so that the two haplotypes can be collapsed into a single haploid representation of the genome sequence (Fig. 2a). Accordingly, inbred lines were used for the first plant genome sequences, Arabidopsis and rice (Arabidopsis Genome Initiative 2000; International Rice Genome Sequencing Project 2005).

To minimize the bioinformatic challenges of heterozygosity in cassava, the reference genotype was chosen to be the inbred accession AM560-2. This accession was generated by Hernan Ceballos at the International Center for Tropical Agriculture (CIAT) by repeatedly selfing MCol1505, a Colombian cassava with good agronomic performance and excellent cooking quality (H. Ceballos, *personal communication*). As the product of three generations of selfing (S3), an estimated 94% of the AM560-2 genome is homozygous (Bredeson et al. 2016). In the reference genome sequence, the remaining heterozygous 6% of the genome (distributed across 10 long blocks larger than 1 Mb) is represented as an alternating mosaic of the two haplotypes.

In typical usage, a reference genome serves as a unifying platform for studying the gene content, gene expression, and genetic variation of a species, and provides common reference coordinates for labeling genes and genetic variants. Since the chromosomes of an outbred, sexual species are continually recombining with one another, there is no “special” genotype, and any haploid reference is generally as good as any other for the purpose of developing genetic markers, aligning genetic data from other individuals, and studying gene expression through methods such as RNA-seq. Thus the AM560-2 reference has general utility as an organizing platform, and has even been used to analyze sequence from other species like *M. glaziovii* (Bredeson et al. 2016). The primary drawback to having a single reference arises from insertion-deletion-duplication variation at the scale of hundreds to thousands of bases. If the reference genotype has a deletion relative to other members of the species, or if other members have an insertion, the corresponding sequence data from those individuals may not align to the reference, since it does not include the relevant locus. This drawback can be addressed by expanding the concept of a reference genome sequence to a “pan-genome” that seeks to represent all the common structural variants in a species (Gao et al. 2019; Gordon et al. 2017; Li et al. 2014; Zhao et al. 2018; Hirsch et al. 2014).

The AM560-2 cassava reference genome has seen four major releases, evolving along with developments in genome sequencing technologies and algorithms. This evolution is summarized in Table 1, which shows quantum improvements based on several metrics for genome assembly quality. The ideal assembly would capture the entirety of every chromosome, from telomere to telomere, without any gaps. In practice, transposable elements and other repetitive sequences scattered throughout the genome are difficult to resolve. Assembling repetitive sequence is especially problematic in the extended pericentromeric regions of cassava (Bredeson et al. 2016). Long and accurate reads are useful for overcoming these challenges, since small differences between repeated units, or variation in flanking sequence, can be used to bioinformatically assign each distinct repetitive copy to a unique position in the genome sequence. However, in the absence of a read that passes through a repetitive or otherwise complex region, current practice is to link the sequence on either side of the repetitive region, placing a string of “N”s in between to signify the unknown sequence. This is known as a gap in the assembly. Depending on the linking or “scaffolding” technology used to span gaps, it is often possible to estimate gap sizes. For a detailed review of contemporary approaches to plant genome assembly, see (Michael and VanBuren 2020).

**Fig. 3:**
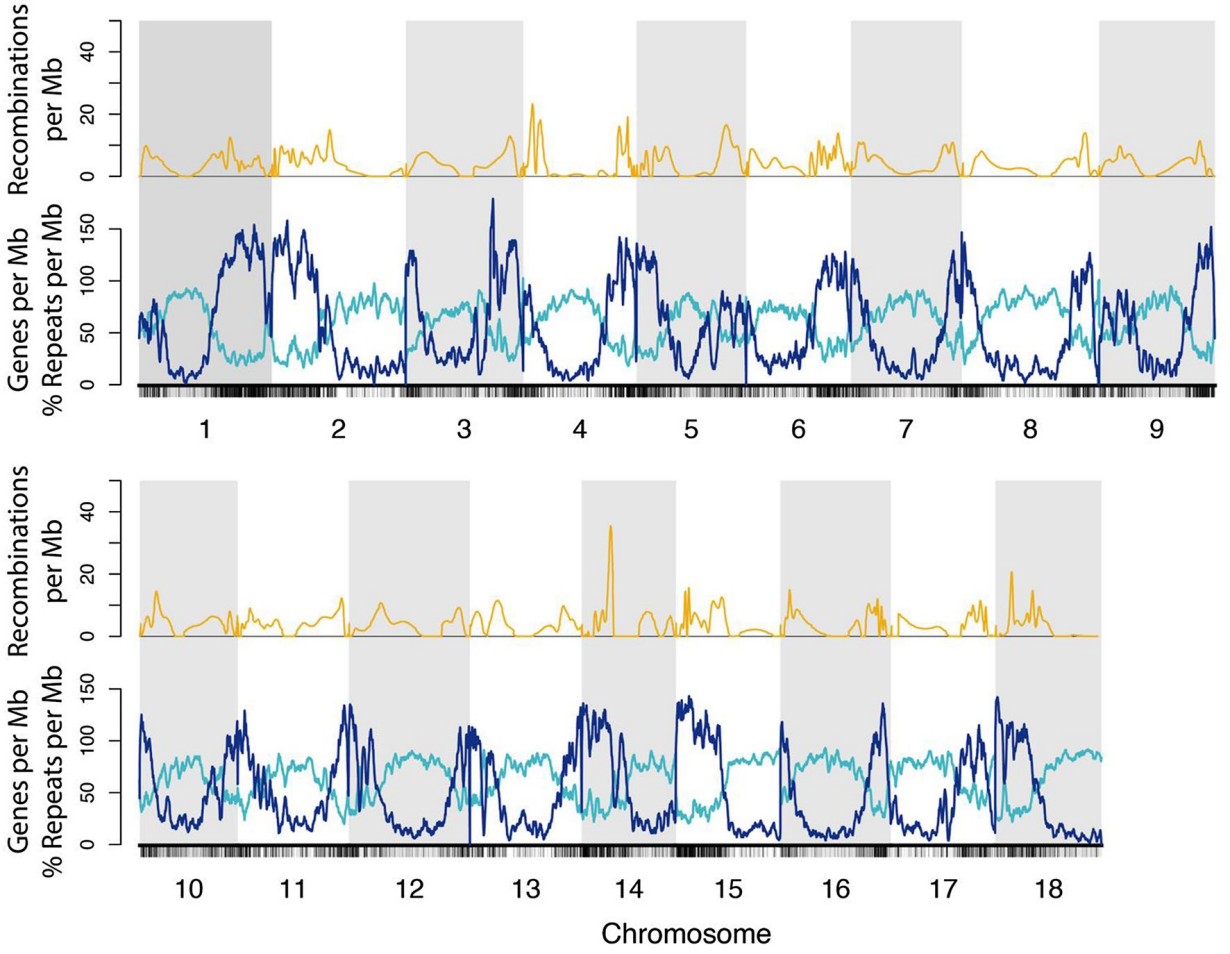
Repeats, genes, and recombination frequency in the AM560-2 v7 cassava genome. Repeat density (light blue lines), gene count (blue lines), and recombination rate (gold lines) are plotted. Genic regions are anticorrelated with repetitive regions (Y-axis). Regions with low recombination frequency tend to co-occur with areas of high repeat density, thus, these hard-to-assemble regions also tend not to benefit from scaffolding information provided by a genetic map. Repeat density is measured as the fraction of bases that are annotated as repetitive in 1 Mb sliding windows sampled every 100 kb along the AM560-2 v7 chromosomes. The gene count was also taken with 1 Mb sliding windows every 100 kb. Recombination rate is measured as the number of recombinations per 1 Mb sliding window (100 kb step) using the first derivative of a natural cubic spline-smoothed fit line to the ICGMC 2014 framework map anchored to the v7 genome sequence. The marker positions of the framework map are plotted with vertical black ticks below the X-axis

Contiguous sequences free of gaps are called “contigs,” and sequences containing gaps are referred to as “scaffolds” or “super-contigs.” A measure of the typical length of a sequence is the “N50 length,” which is the weighted median contig length of assembled sequence, that is, the median number of bases between gaps in the assembly (Lander et al. 2001). Thus, it is a measure of assembled sequence contiguity. Table 1 shows that the typical length of contiguous sequence between gaps has grown by a factor of 60 between the 2009 and 2019 releases and is now nearly 700 kilobases (kb). Similarly, the total contig length has grown by 50%, reflecting the greater coverage of repetitive regions within the latest reference genome. The number of predicted genes has largely remained stable at ~ 30–33k, although this metric obscures the considerable improvements in the quality of individual gene predictions, which are now bolstered by extensive transcriptomic data developed over the past decade, as well as interspecific comparisons that take advantage of sequence similarity between encoded proteins across plant species. The stability of the gene number, despite dramatic improvements in contiguity and total sequence, implies that the major improvements have come in resolving repetitive sequences. This interpretation is supported by the anticorrelation between genes and repeats, with genes clustered near the distal ends of chromosomes and repetitive elements clustered in extended pericentromeric regions (Fig. 3).

A major development in the evolution of the cassava reference genome was the construction of a dense genetic map, which allowed assembled sequences to be organized into chromosome-scale sequences (these are sometimes referred to as “pseudomolecules”) (ICGMC 2014). Smaller scaffolds that are not situated into chromosome-scale scaffolds are often called “unplaced.”

The first cassava reference genome, released in 2009 as version 4.1 (v4.1), was constructed using the Roche 454 sequence technology, in a collaboration led by Steve Rounsley, Dan Rokhsar, Chinnappa Kodira, and Tim Harkins, with support from the Bill and Melinda Gates Foundation, the U.S. Department of Energy Joint Genome Institute, and 454 Genomics. Although fragmented, it assembled the vast majority of gene space (Table 1) (Prochnik et al. 2012) and provided a first look at the repeat composition of the cassava genome. Version 5 (v5) improved upon the v4 assembly by elevating it to chromosome scale, ordering and orienting 57% of the v4 assembled sequences into n = 18 chromosomes by taking advantage of a dense composite genetic map ((ICGMC 2014; De Carvalho and Guerra 2002). With the advent of new sequencing technologies, the version 6 (v6) assembly of AM560-2 started afresh from deep (120×) Illumina short-read sequence assembled *de novo* into contigs. These contigs were ordered and oriented into scaffolds with Illumina mate pairs and fosmid ends, and then to chromosome scale using genetic maps (Bredeson et al. 2016). V6 represented an advance over v5 by capturing 18% more total contig sequence, increasing overall sequence contiguity, and incorporating 45% more sequence into chromosomal scaffolds. It has served as the cassava community’s primary reference platform for genomic analyses since its release (Bredeson et al. 2016; Kuon et al. 2019; Wolfe et al. 2019; Kayondo et al. 2018; Nzuki et al. 2017; Ramu et al. 2017; Andrade et al. 2019).

The version 7 (v7) reference assembly was released on Phytozome in 2019. This latest assembly incorporated PacBio continuous long read (CLR) sequences, most longer than 10 kb. Long reads more effectively traverse genomic repeats, allowing them to be captured in assembled contigs more completely. In contrast, the assembly of short reads, e.g. Illumina, often leads to the fragmentation or collapse of repetitive sequences, as their sequence similarity with many other loci in the genome renders them refractory to assembly. Raw CLR data suffer from an order-of-magnitude higher error rate than Illumina, but modern computational methods have largely overcome this issue (Chin et al. 2013). The Canu assembler (Koren et al. 2017) was used to construct *de novo* contigs from error-corrected CLR data 4 kb or longer, constituting 34× depth coverage, which were scaffolded using an approach similar to that of v6. The use of long reads facilitated an increase of over 100 Mb of included sequence, and over 25× increase in contiguity. We note that while the current 669 Mbp reference assembly is somewhat shorter than the 750 Mbp haploid genome size estimated for other genotypes by flow cytometry (Kuon et al. 2019), it is close to the size estimated directly from shotgun sequencing. Any missing sequences are likely to be confined to highly repetitive regions, since genic regions are essentially completely assembled within v7. The v7 assembly is available at Phytozome and Cassavabase, as discussed below.

Version 8 (v8), currently being assembled at UC Berkeley, will combine High-throughput Chromatin Conformation Capture (Hi-C) data with the contigs and long-range linking information used to assemble v7. Hi-C captures DNA-DNA contact pairs between folded DNA sequences within the cell nucleus, and two closely linked loci along the same linear chromosome are more likely to be in contact than two distant loci (Lieberman-Aiden et al. 2009). Thus, Hi-C data can be used to place DNA sequences relative to one another during genome assembly (Burton et al. 2013; Putnam et al. 2016; Dudchenko et al. 2017). For cassava, Hi-C is useful for incorporating additional contigs/scaffolds into chromosomes, and refining contig/scaffold orders and orientations, especially in areas of low recombination rate and for small scaffolds, where the genetic map does not have much resolution (Fig. 3).

Researchers who have investigations underway using a given version of a genome assembly may feel frustrated to hear that a new one is being released. Must all the analyses be redone using the new assembly? For many purposes (e.g., QTL mapping, or RNAseq analysis) the cassava v6 reference is satisfactory. There are, however, genomic regions that are fragmented or even absent in the earlier assemblies, so it is in general worth examining loci of interest in the newer assembly. It’s important to note that a given gene may be annotated or named differently in different genome assembly versions due to improvements in the completeness and correctness of the underlying assembly, the complement of expression- and homology-based evidence supporting it, and the computational algorithms used to evaluate and integrate that evidence for modeling that gene.

## Additional cassava genome assemblies

While the AM560-2 reference genome is a starting point for cassava genomics, increasingly the sequencing of additional cassava diversity is beginning to provide a broader perspective on cassava genetic variation. Wang and colleagues reported the assembly of two cassava varieties, W14 and KU50 (Wang et al. 2014). The former was reported to be *M. esculenta* ssp. *flabellifolia* (Pohl) Cif and is referred to as a “wild cassava” or “wild ancestor”. Subsequent analysis using the sequence reads deposited by Wang et al., however, showed that the plant sequenced as W14 does not belong to *M. esculenta*, but is more closely related to *M. glaziovii* (Bredeson et al. 2016). Including this accession in a phylogenetic analysis derived from a broader selection of *Manihot* could help determine whether it is an *M. glaziovii* or a member of another *Manihot* species. KU50 is an improved cassava variety popular in Southeast Asia (Ceballos et al. 2020). The W14 and KU50 assemblies were constructed from Illumina whole genome shotgun short-insert and mate-pair libraries, 454 sequencing of BAC clones, and long-range linkages from the AM560-2 reference genome. The W14 and KU50 assemblies are important contributions to our understanding of *Manihot* genomic diversity but are less complete than the subsequently released AM560-2 reference v6 and v7. These later sequences are therefore recommended as the current best references for mapping short read datasets for genotyping purposes.

The advent of long-read sequencing technologies makes it possible to tackle outbred genomes and, in principle, to separately assemble each haplotype with the aim of recovering all 2n = 36 chromosomes of a diploid cassava. Important first steps in this direction were taken by Kuon and collaborators (Kuon et al. 2019). These authors sequenced the African varieties TME3 and 60444, selected for their resistance and susceptibility, respectively, to cassava mosaic disease (CMD). The genomes were assembled with the Canu v. 1.4 assembler, IrisView, and HiRise software using a combination of PacBio CLR reads (70× coverage), Bionano optical mapping, and Hi-C technologies. The resulting chromosomal scaffolds were deposited in NCBI (Table 2).

**Table 2:** Accessing key cassava genomic resources

Resource	Portal	url
AM560-2 v6.1	Phytozome	https://phytozome-next.jgi.doe.gov/info/Mesculenta_v6_1
AM560-2 v7.1	Phytozome	https://phytozome-next.jgi.doe.gov/info/Mesculenta_v7_1
W14 assembly (unresolved Manihot)	NCBI	https://www.ncbi.nlm.nih.gov/assembly/GCA_000737105.1
KU50 assembly	NCBI	https://www.ncbi.nlm.nih.gov/assembly/GCA_000737115.1
TME3 assembly	NCBI	https://www.ncbi.nlm.nih.gov/assembly/GCA_003957995.1
60444 assembly	NCBI	https://www.ncbi.nlm.nih.gov/assembly/GCA_003957885.1
HapMapI SNV calls (download)	Phytozome	https://genome.jgi.doe.gov/portal/pages/dynamicOrganismDownload.jsf?organism=Mesculenta, under Mesculenta → v6.1 → diversity
Genomic and phenotypic data and breeding metadata	Cassavabase	https://cassavabase.org/
ICGMC genetic map viewer	Cassavabase	https://cassavabase.org/cview/map.pl?map_id=2
HapMapII sequence and SNP data	Cassavabase	ftp://ftp.cassavabase.org/HapMapII/
HapMapII SNPs (browser)	Cassavabase	https://cassavabase.org/jbrowse_cassavabase/?data=data%2Fjson%2Fhapmap_variants
Bart Lab Cassava Atlas	none	http://shiny.danforthcenter.org/cassava_atlas/

A major challenge for “diploid” genome assembly of heterozygous genomes is separating haplotypes whose sequences can be very similar. The ultimate goal of “diploid” genome assembly is the generation of complete phased sequences for both haplotypes, i.e., all 2N chromosomes of a diploid. For TME3, Kuon et al. make considerable progress towards this goal, and report the assembly of a “primary” haplotype totaling 732 Mbp of contig sequence, close to the genome size estimated by flow cytometry. They also assemble over 200 Mb of alternative haplotype sequence, providing direct access to allelic variation in this important cultivar, with similar results obtained for 60444. To attempt to link these contigs into phased chromosomal haplotypes, Kuon et al. generated high coverage optical maps and HiC chromatin data for both accessions, a first for cassava. Kuon et al. describe two assemblies derived from these data. The first, a “primary” assembly deposited in NCBI (Table 2), combined contigs with optical map and chromatin data to obtain a composite assembly that mixed both primary contigs and alternate haplotype contigs. This assembly contains 300 Mb of “gap” sequence represented by strings of “N”s, where the length of the gap is estimated using the optical map. Comparison of the deposited assemblies from Kuon et al. with the AM560-2 reference shows that some gaps appear to arise from the artifactual interaction of optical map and HiC scaffolding (Bredeson, unpublished). For example, haplotypes may be juxtaposed, with gaps from the bionano assembly of each haplotype retained. So, a sequence that looks like A1-B1-C1-D1 and A2-B2-C2-D2 in the two haplotypes may be represented as A1-NN-C1-D2-NN-B2 in the primary assembly, creating both artifactual gaps as well a local mis-orderings. Thus, in many cases the “gaps” in this sequence are not truly missing but may be misplaced nearby.

To remove these artifacts, Kuon et al. performed additional processing to remove residual duplicate sequences and gaps using tools such as Purge Haplotigs (Roach et al. 2018). The resulting final assemblies represent mosaics of the two haplotypes of each accession, rather than complete haplotype-resolved diploid assemblies, but still capture the genetic structure of these two important cultivars and appear to capture additional repetitive sequence not found in the AM560-2 reference. We note, however, that the final pseudomolecules described by Kuon et al. total 796 Mb for TME3 and 854 Mb for 60444, are longer than the estimated 750 Mb from flow cytometry (Kuon et al. 2019), presumably due to undetected residual duplication. These assemblies supported the analysis of gene families that had differentially expanded and diverged among TME3, 60444, and AM560, including genes involved in antiviral and other disease response processes. Developing methods for accurately and efficiently performing diploid assembly is an area of ongoing and rapid development in bioinformatics, and we expect that future “diploid” assemblies of cassava and other outbred crops will become commonplace in the next few years. These assemblies will capture both haplotypes at high contiguity and with phase accuracy (i.e., even distant segments of a given maternal or paternal chromosome will be on the same reported pseudomolecule sequence). These will be particularly useful for detailed examination of specific loci (e.g., disease resistance, quality traits) and the genetic variation at such loci across germplasm, to guide both breeding and engineering of improved cassava, as well as the exploration of larger structural variation across cassava and related species.

As more and more genomic variation is discovered in cassava, it will be a challenge for users to take advantage of these resources. For example, a diploid genome sequence is not ideal for mapping resequencing data from other accessions, since reads from the target accession will not have a unique locus to map to but can align more-or-less equally well to either haplotype of the diploid genome sequence. We therefore expect that a high-quality haploid reference will continue to be important as an organizing principle for this flood of genome data. Even in genomic regions with structural variants (e.g., insertions, duplications, inversions) not found in the reference, it will be important to locate these on the reference genome and with regard to markers on the genetic map, providing a common coordinate system. Following the lead of the human genome we anticipate the development of collections of alternate haplotypes (as done for example for the highly polymorphic human HLA region) and ultimately a graphical “pan-genome” representation of the collection of diploid genomes that can be directly queried by users. Such tools are still in development but given the genomic sophistication of the cassava research and breeding community we anticipate their rapid adoption.

## Resequencing and Cassava Genetic Diversity

In addition to *de novo* assembled genomes, many cassava varieties have been ‘re-sequenced’ using short read sequencing technologies. Here we use re-sequencing to mean that sequence was generated with a uniformly-random sampling approach from across the genome of a cultivar of interest (whole genome shotgun [WGS]), but not assembled *de novo*. Such shotgun sequences can be aligned to the reference to learn about the genome of the re-sequenced variety. Comparisons between re-sequenced individuals can characterize family-level relatedness and larger-scale population structure. In the HapMapI study, Bredeson *et al.* performed whole genome resequencing of 53 *M. esculenta*, mostly cultivated cassavas, and five other *Manihot* (Bredeson et al. 2016). It can be challenging to track down reliable information for a given cassava variety. The HapMapI team compiled provenance/pedigree, descriptive, and/or phenotype information, as available, for the accessions they re-sequenced or genotyped. This information is included in Supplemental File 1, along with identifiers for obtaining the raw sequence from the NCBI Sequence Read Archive (SRA). The HapMapII project incorporated the WGS data from HapMapI and added more diversity, bringing the total to 241 total accessions, comprising 203 cultivated cassavas plus hybrids and wild relatives (Ramu et al. 2017). The raw sequence data generated for HapMapII are available for download on Cassavabase, along with the single nucleotide polymorphism (SNP) data for all 241 accessions (See Table 2).

## High throughput genotyping technologies

In the cassava genomics era, there has been a shift from using individual or sparse markers, such as simple sequence repeats (SSRs), to generating and using sets of markers derived from SNPs, which are denser in the genome. SNP-based approaches have included EST-derived marker genotyping arrays, such as KBiosciences KASPar arrays and a 1536 SNP Illumina GoldenGate array (Ferguson et al. 2012a, b; Rabbi et al. 2012; Ferguson et al. 2019), and reduced representation approaches, such as genotyping-by-sequencing (GBS) (Elshire et al. 2011; Hamblin and Rabbi 2014). GBS has been applied broadly in the cassava genomics community (Rabbi et al. 2014a, b; ICGMC 2014; Bredeson et al. 2016; Wolfe et al. 2016a, b; Rabbi et al. 2017; Kayondo et al. 2018; Esuma et al. 2016). Today, researchers can perform GBS in-house or use a fee-for-service GBS facility; DArTseq is a similar technology offered as fee-for-service (Diversity Arrays Technology Pty, Canberra, Australia). Both GBS and DArTseq rely on the same principle of using restriction enzyme digestion paired with Illumina sequencing to sample a reduced―yet broadly distributed and repeatable―fraction of the genome. SNPs are identified, and genotypes called, from the resulting sequence reads. DArTseq service includes this analysis, returning reports with genotype calls for each individual across a set of loci. Several computational approaches for analyzing GBS data have been used in cassava, e.g. TASSEL/TASSEL-GBS (Bradbury et al. 2007; Rabbi et al. 2014a, b; Glaubitz et al. 2014; Ramu et al. 2017), and the pipeline implemented by the International Cassava Genetic Map Consortium (ICGMC 2014).

The HapMapI project used GBS to genotype 268 African cassavas, and the SRA identifiers are listed in the paper’s Supplementary File 1 (Bredeson et al. 2016). These data were demultiplexed (sorted by sample) and the GBS-facilitating sequences removed, so that the deposited sequence reads contain only the cassava sequence.

## Genetic maps

Cassava researchers have been making genetic maps for over 20 years (Fregene et al. 1997). Since 2012, a series of papers have demonstrated the utility of SNP markers for genetic mapping in cassava, especially when combined with phenotype data to map important traits (Soto et al. 2015; ICGMC 2014; Rabbi et al. 2014a, b; Rabbi et al. 2012). The International Cassava Genetic Map Consortium used GBS data from 10 mapping populations to construct a dense composite reference map, comprising 22,403 genetic markers organized into the expected 18 linkage groups (ICGMC 2014). This composite map has ongoing utility, including for improving or evaluating new genome assemblies (ICGMC 2014; Kuon et al. 2019; Bredeson et al. 2016). From version 5 onward, the chromosomes of the AM560-2 reference assemblies have been numbered according to the composite map, e.g. chromosome 7 corresponds to linkage group VII.

## Omics datasets and data access

Many transcriptomics datasets have been published for cassava. Collectively, these datasets describe gene expression patterns across abiotic and biotic stress (Rauwane et al. 2018; Muñoz-Bodnar et al. 2014; Amuge et al. 2017; Zhang et al. 2015; Ding et al. 2016; Vanderschuren et al. 2014; Cohn et al. 2014, 2016) and across different cassava tissue types (Wilson et al. 2017). These transcriptomics datasets have also fueled a significant number of manuscripts characterizing diverse classes of proteins (Hu et al. 2016a, b; Liao et al. 2016; Wei et al. 2016; Ou et al. 2018; Li et al. 2017; Fan et al. 2016; Hu et al. 2015). As of November 17th, 2019, there were 463 *Manihot esculenta* transcriptomic samples available through the SRA run selector tool; a subset of these are organized into 23 BioProjects. A high-quality reference genome is also valuable for proteomics research. For example, Vanderschuren et. al. analyzed proteomic changes that occur during post physiological deterioration (PPD) (Vanderschuren et al. 2014), Wang et al. reported proteomics analysis of cassava root formation (Wang et al. 2016) and multiple groups have characterized the epigenetic profile of cassava by mapping bisulfite sequencing (BS-seq) data onto the cassava reference genomes (Wang et al. 2015; Zou et al. 2017). Application of these diverse omics datasets is described below in the “Applications of genomics” section.

Making genomics data accessible and easily visualized to researchers is challenging. Genomics data are famously large and this presents challenges for storage and processing. In addition, most platforms, once constructed, are rigid and can only be used for the intended analysis types. Currently, the cassava community has several points of entry for accessing genomics data. First, the National Center for Biotechnology Information (NCBI) hosts genomics data from all organisms and provides a suite of tools for query and analysis (described within the handbook here: (The NCBI Handbook 2013)). Phytozome is a plant specific public repository for comparative genomics funded through the Department of Energy (https://phytozome-next.jgi.doe.gov/) and has previously been reviewed (Goodstein et al. 2012). *M. esculenta* references v6.1 and v7.1 and their annotations are available in Phytozome as browsers and for download. The v6.1 browser also contains tracks for single nucleotide variants (SNVs) and indels identified in the 60 *Manihot* analyzed in the HapMapI project (Bredeson et al. 2016), and methylation and RNAseq data from accession TME7 (Wang et al. 2015). See Table 2 for how to access these data in Phytozome.

In addition to the traditional NCBI and Phytozome portals, new platforms dedicated to the cassava scientific community have been developed. Cassavabase is a genomic-based breeding database. As such, it contains comprehensive management and analysis tools for genomic, genotypic, phenotypic (cassava trait ontology) data and breeding metadata. The system implements a “digital ecosystem”, which allows all breeding processes to be tracked in the database and all breeding data to be collected digitally. Cassavabase manages accessions, pedigrees and seed lots, calculation of field layout randomizations, collection of phenotypic data using tablets and other methods such as drones, collection of crossing data, as well as DNA samples for genotyping. Breeders use it to design their field layouts, barcode the field using built-in barcode tools, collect phenotypic and genotypic data from the plants, and plan and track their crossing experiments. Cassavabase can be used to implement genomic selection (GS) via the SolGS tool (Tecle et al. 2014): phenotypic and genotypic data can be correlated and phenotypic predictions generated on lines that have been genotyped, but not phenotyped. By leveraging previous phenotyping rather than conducting it each time, these genomic predictions accelerate the breeding cycle by a significant factor, and thus increase genetic gain per unit time. Cassavabase currently contains over 30 years’ worth of experimental data, across 250 locations in a dozen countries, including 258 phenotypic traits. Researchers use Cassavabase to explore or analyze various genomic resources by using tools such as BLAST or by identifying new trait-associated genome regions using tools such as GWAS. Cassavabase contains user friendly applications and visualization tools for genomic resources, such as a pedigree viewer, the ICGMC genetic map viewer, and a browser showing the SNPs identified by the HapMapII project (see Table 2). High (GBS) and low density (ie: Kompetitive allele specific PCR, KASP) genotyping assay data are available for more than 30,000 accessions. Users who wish to submit newly re-sequenced resources information may do so using the “Sequencing Status” feature on each germplasm page. All aforementioned functionalities are publicly available and can be accessed by creating a free user account.

For genomics analyses and visualization other than those described above, most scientists rely on lab specific tools and while these methods are reported during publication, this system does not facilitate subsequent queries of the data by future researchers, especially those without a strong computational skillset. The makers of Rstudio (Copyright 2017 Rstudio Inc.) developed a way to turn a now commonly used, open-source data processing language, R (R Core Team 2014), into interactive web applications. They call this development R Shiny (Chang et al. 2017)). This development makes it easy for intermediate and expert R users to create web-ready applications for data queries and visualization. Shiny, just like all R packages, is a large collection of tools and functions that can be pieced together to create a fully functioning website. Wilson et al. used this platform to create a web-based interface, Bart Lab Cassava Atlas, for exploring tissue specific gene expression patterns for cassava (Wilson et al. 2017). Recently, they added data from a previous paper (Cohn et al. 2014), to this tool and in the future, it may be possible to further expand this platform with the additional datasets described above.

## Applications of genomics

When a reference genome is published, the authors may draw attention to specific aspects of the genome or use the newly available resource for a specific purpose. In the case of the cassava reference genome, the first scientific manuscript describing the genome assembly accompanied v4.1 (Prochnik et al. 2012). This manuscript communicated the details of this resource to the growing cassava community and was quickly incorporated into additional research efforts. Many papers have used the reference genome to investigate a specific class of proteins. For example, Lozano et al. (2015) used cassava gene annotations from v4.1 (Prochnik et al. 2012) to catalog the NBS-LRR disease resistance genes, and a subsequent chromosome-scale assembly, v5.0, (ICGMC 2014) to show that this class of genes are arranged in clusters in the genome. Similarly, WRKY transcription factors, potassium ion (KUP) transporters (Ou et al. 2018), calcium-dependent protein kinases (Hu et al. 2016a, b), bZIP and ERF transcription factors (Hu et al. 2016a, b; Fan et al. 2016), to name a few, have been the foci of recent papers. This resource has also been used for gene discovery. For example, Cohn and Bart et al. used the reference genome to identify cassava genes upregulated during attack by a bacterial pathogen (Cohn et al. 2014). In another transcriptomics focused study, Amuge et al. investigated genes that were differentially expressed between susceptible and tolerant cassava varieties during attack by Ugandan Cassava Brown Streak Virus (Amuge et al. 2017). The *M. esculenta* genome is paleotetraploid, i.e., is the result of an ancient genome duplication (Bredeson et al. 2016). Thus, in conducting these types of analyses, researchers should be aware that the cassava genome may contain two copies (paralogs) of a gene of interest. For example, paralogs *CYP79D1* and *CYP79D2* both code for the enzyme CYP79D, a member of the cyanogenic glucoside biosynthetic pathway (Andersen et al. 2000).

A major goal of the HapMapI project was to characterize global cassava diversity. The authors leveraged their reference genome assembly v6.1 as the platform for cataloging SNVs in 60 resequenced *Manihot* accessions, including diverse cassava varieties, putative progenitor species, wild relatives; and 268 African cassavas (Bredeson et al. 2016). Major findings included introgressed *M. glaziovii* segments in many cassava accessions, particularly in improved varieties, and widespread relatedness among cultivated cassavas. In evaluating relatedness among the cassavas via identity by descent, they showed that some cassavas considered to be different accessions are clones of one another, notably TME3, TME14, and TME7; and TME204 and TME419. Here, this means there is no more parsimonious way to explain their high degree of relatedness (sharing > 98% of variable sites relative to the reference) than that they are related via clonal propagation. Measured differences must be due to genotyping error or somatic mutations acquired over time since cloning. Phenotypic differences between clonally related accessions, then, may be due to somatic mutation or epigenetic changes.

The HapMapII project used reference assembly v6.1 with the WGS data from 241 accessions to reveal that cultivated cassava varieties harbor a substantial burden of deleterious mutations (genetic load) maintained in the heterozygous state (Ramu et al. 2017). Subsequent QTL and GWAS studies showed that loci associated with resistance to cassava brown streak disease (CBSD) overlap with regions of *M. glaziovii* introgression (Nzuki et al. 2017; Kayondo et al. 2018). Rabbi et al. showed that a region associated with both dry matter content and carotenoid content coincides with an introgressed region on chromosome 1, which also has elevated linkage disequilibrium (LD), indicating reduced recombination frequency (Rabbi et al. 2017). Leveraging the WGS data from HapMapI/II, and GBS data from several cassava breeding programs, Wolfe and colleagues identified introgressed *M. glaziovii* segments in over 2700 cassavas, with American and African populations showing on average 2% to 4.2% of *M. glaziovii* introgression, genome wide. A reduced level of recombination was observed in the introgressed regions on chromosomes 1 and 4. When heterozygous, the introgressed segments are associated with preferred traits such as CBSD resistance and higher dry matter content, but over progressive rounds of genomic selection homozygotes are selected against. Carrying these haplotypes in the heterozygous state, thus also plays a role in the maintenance of genetic load (Wolfe et al. 2019).

Genomics tools have also helped narrow the genomic location of important loci, such as those that confer resistance to Cassava Mosaic Disease, and the major loci for root carotenoid and dry matter content (Wolfe, Rabbi, et al. 2016a, b; Rabbi et al. a, b, 2017). In addition to gene discovery, high quality genomics resources are invaluable for new genome editing technologies. The design of CRISPR-Cas9 guide sequences, for example, relies on a good quality genome assembly and accurate annotation of gene models. Where available, accession-specific re-sequencing data may be used to ensure that the target sequence matches the guide and to predict potential off-targets. In a proof of principle study for genome editing in cassava, Odipio and colleagues (Odipio et al. 2017) disabled *Phytoene desaturase*, resulting in the expected albino phenotype (Odipio et al. 2017). This technology is also being deployed to create agriculturally important phenotypes. For example, Gomez and Lin *et al.* showed that disabling *nCBP-1* and *nCBP-2* conferred resistance to CBSD (Gomez et al. 2019). A team from ETH Zurich generated waxy (amylose-free) cassava by mutating the GBSS gene, and reduced amylose cassava by editing PTST1 (Bull et al. 2018). In the first report of a knock-in at an endogenous cassava locus, Hummel and colleagues used genome editing to generate glyphosate tolerant plants by swapping the promoter and changing two amino acids of the EPSPS gene (Hummel et al. 2018).

Notably, the rate of published research articles that use existing cassava -omics datasets is increasing overtime, demonstrating the need for such resources and reflecting both an increase in the number of researchers interested in cassava and an increased comfort with using these resources among the cassava community. Cassava has become a model for how to bring an orphan crop into the genomics era. We look forward to further democratization of both the generation and the usage of cassava genomic resources.

## Future perspectives

The term “finished” is hardly ever applied to genome assemblies, and rightly so. With the exception of the simplest viral and bacterial genomes, even the highest quality genome sequences are incomplete and a constant focus for further improvement. With this in mind, the cassava community eagerly awaits new genome assemblies including the release of the AM560-2 v8 assembly. This assembly should be the most complete and accurate version available, particularly in centromeric and distal subtelomeric regions.

In addition to increased contiguity of genomic sequence, we can look forward to fully resolved haplotypes for heterozygous genomes such as cassava. Advancements in sequencing technologies have reduced the costs and improved the capacity for sequencing and assembling genomes. As more non-model plants enter the genomic era, it is becoming evident that the utility of haploid genome assemblies is limited in some cases. This is especially problematic in plants with high heterozygosity and strong inbreeding depression, where the generation of homozygous lines for sequencing may be impossible and with limited relevance to the natural state of these genomes. These haploid assembly strategies collapse both haplotypes into a single sequence representing a pseudo-haploid reference (Fig. 2b). During this process of collapsing the haplotypes, assembly errors may be introduced, including the generation of variants which may not be present in either haplotype (Church et al. 2015).

In an effort to overcome these challenges, recent advancements in genome-phasing algorithms have been developed. The trio binning (Koren et al. 2018) approach is the current gold standard for genome phasing. While effective and accurate, this approach relies on the ability to sequence a trio, including two parents and their progeny. This is prohibitive in some cases, such as sequencing farmer preferred cassava lines, where the parents are often unknown. Furthermore, due to the need to sequence long reads from the sample of interest, as well as short reads from the parental lines, this method is more resource input intensive. Using the information from the sequencing of the parents, this approach bins maternal and paternal sequence reads of the progeny prior to their assembly. Thus, each haplotype is then assembled independently and accurately.

In lieu of parental material, other approaches have been developed for phased assembly. The FALCON/FALCON-Unzip and Hifiasm tools attempt to separate phases during the assembly process by identifying and extracting regions containing polymorphisms and structural variants (Chin et al. 2016; Cheng et al. 2020). The output of the FALCON/FALCON-Unzip algorithms is an assembled primary contig alongside “haplotigs” representing a corresponding heterozygous assembly for a specific region. These algorithms, however, have difficulty in resolving regions of high haplotypic divergence (> 4%) (e.g. Padgitt-Cobb et al. 2019) and thus assemble both haplotypes in the primary assembly, similarly to that described in (Kuon et al. 2019). Other orthogonal tools have been developed to facilitate the haplotig purging process (Roach et al. 2018; Guan et al. 2020) and can help resolve the haplotypes. However, this *de novo* approach does not fully resolve phase switching errors, in which sequences from different haplotypes are assembled adjacent to one another. By utilizing information from long range genomic sequencing (Hi-C), a new algorithm (FALCON-Phase, (Kronenberg et al. 2018)) attempts to resolve these errors and can be applied iteratively on both contigs and scaffolds, thus allowing resolution of chromosome level phases. The end result is two assembly files which represent an approximate depiction of the two haplotypes. This phasing approach has been successfully demonstrated for the human genome, as well as several heterozygous animal species and an Arabidopsis F_1_ hybrid (Kronenberg et al. 2018). It thus might also be applicable for genome assembly of plant species with high heterozygosity, such as cassava.

Structural variations are likely important to cassava genomic studies and breeding but are not easily detected using short read resequencing. With the availability of long read technologies such as PacBio and BioNano, it may now be possible to survey cassava structural variations by sequencing and assembling many accessions, as is being done in other crops such as tomato (Alonge et al. 2020). Lack of concordance between physical positions of two haplotypes is caused by large structural variations and this can complicate downstream analyses. For example, genes might not directly align between the haplotypes. This can also cause difficulty when comparing mapping results from GBS for example, as SNPs will not land in exact physical positions on both phases. New browsers need to be designed to allow users to explore this type of structural variation (Garrison et al. 2018). The ability to resolve this information is crucial for accurate understanding of genetics in these species and further identification of important genes and variants which may have been masked in a collapsed haploid assembly. Cassava’s high genetic load and clonal propagation have maintained the highly heterozygous nature of its genome for generations. While the technologies and approaches described in this review have provided valuable new insights into cassava structural genomics, in the near term, researchers may be best served by combining these genomics approaches with more standard techniques such as sequence capture and sequencing of bacterial artificial chromosome (BAC) clones to fully resolve particularly important genomic locations. In conclusion, by employing old and new genome phasing strategies we are poised to make valuable gains in understanding of cassava evolution, domestication and breeding.

## References

[CR1] Alonge M, Wang X, Benoit M, Soyk S, Pereira L, Zhang L, Suresh H (2020). Major impacts of widespread structural variation on gene expression and crop improvement in Tomato. Cell.

[CR2] Amuge T, Berger DK, Katari MS, Myburg AA, Goldman SL, Ferguson ME (2017). A time series transcriptome analysis of cassava (*Manihot esculenta* Crantz) varieties challenged with ugandan cassava brown streak virus. Sci Rep.

[CR3] Andersen MD, Busk PK, Svendsen I, Møller BL (2000). Cytochromes P-450 from Cassava (*Manihot esculenta* Crantz) catalyzing the first steps in the biosynthesis of the cyanogenic glucosides linamarin and lotaustralin. Cloning, functional expression in *Pichia pastoris*, and substrate specificity of the isolated recombinant enzymes. J Biol Chem.

[CR4] Andrade LRB, Sousa MBE, Oliveira EJ, de Resende MDV, Azevedo CF (2019). Cassava yield traits predicted by genomic selection methods. PloS ONE.

[CR5] Arabidopsis Genome Initiative (2000). Analysis of the genome sequence of the flowering plant *Arabidopsis thaliana*. Nature.

[CR6] Bart RS, Taylor NJ (2017). New opportunities and challenges to engineer disease resistance in cassava, a staple food of African small-holder farmers. PLoS Pathog.

[CR7] Bradbury PJ, Zhang Z, Kroon DE, Casstevens TM, Ramdoss Y, Buckler ES (2007). TASSEL: software for association mapping of complex traits in diverse samples. Bioinformatics.

[CR8] Bredeson JV, Lyons JB, Prochnik SE, Albert Wu G, Ha CM, Edsinger-Gonzales E, Grimwood J (2016). Sequencing Wild and cultivated cassava and related species reveals extensive interspecific hybridization and genetic diversity. Nat Biotechnol.

[CR9] Bull SE, Seung D, Chanez C, Mehta D, Kuon J-E, Truernit E, Hochmuth A (2018). Accelerated ex situ breeding of GBSS- and PTST1-edited Cassava for Modified starch. Sci Adv.

[CR10] Burton JN, Adey A, Patwardhan RP, Qiu R, Kitzman JO, Shendure J (2013). Chromosome-scale scaffolding of de Novo genome assemblies based on chromatin interactions. Nat Biotechnol.

[CR11] Ceballos H, Rojanaridpiched C, Phumichai C, Becerra LA, Kittipadakul P, Iglesias C, Gracen VE (2020). Excellence in cassava breeding: perspectives for the future. Crop Breed Genet Genom.

[CR12] Chang W, Cheng J, Allaire JJ, Xie Y, McPherson J (2017) Shiny: web application framework for R. R Package Version 1.0.5 (version 1.0.5). https://CRAN.R-project.org/package=shiny.

[CR13] Cheng, H, Concepcion GT, Feng X, Zhang H, Li H (2020) Haplotype-resolved de novo assembly with phased assembly graphs. http://arxiv.org/abs/2008.01237. 10.1038/s41592-020-01056-5 PMC7961889 33526886

[CR14] Chin C-S, Alexander DH, Marks P, Klammer AA, Drake J, Heiner C, Clum A (2013). Nonhybrid, finished microbial genome assemblies from long-read SMRT sequencing data. Nat Methods.

[CR15] Chin C-S, Peluso P, Sedlazeck FJ, Nattestad M, Concepcion GT, Clum A, Dunn C (2016). Phased diploid genome assembly with single-molecule real-time sequencing. Nat Methods.

[CR16] Church DM, Schneider VA, Steinberg KM, Schatz MC, Quinlan AR, Chin C-S, Kitts PA (2015). Extending reference assembly models. Genome Biol.

[CR17] Cohn M, Bart RS, Shybut M, Dahlbeck D, Gomez M, Morbitzer R, Hou B-H, Frommer WB, Lahaye T, Staskawicz BJ (2014). Xanthomonas axonopodis virulence is promoted by a transcription activator-like effector-mediated induction of a SWEET sugar transporter in Cassava. Mol Plant Microbe Interact MPMI.

[CR18] Cohn M, Morbitzer R, Lahaye T, Staskawicz BJ (2016). Comparison of gene activation by two TAL effectors from xanthomonas axonopodis Pv. manihotis reveals candidate host susceptibility genes in Cassava. Mol Plant Pathol.

[CR19] de Carvalho R, Guerra M (2002). Cytogenetics of *Manihot esculenta* Crantz (cassava) and eight related species. Hereditas.

[CR20] Ding Z, Zhang Y, Xiao Y, Liu F, Wang M, Zhu X, Liu P (2016). Transcriptome response of Cassava leaves under natural shade. Sci Rep.

[CR21] Dudchenko O, Batra SS, Omer AD, Nyquist SK, Hoeger M, Durand NC, Shamim MS (2017). De Novo assembly of the aedes aegypti genome using Hi-C yields chromosome-length scaffolds. Science.

[CR22] Elshire RJ, Glaubitz JC, Sun Q, Poland JA, Kawamoto K, Buckler ES, Mitchell SE (2011). A robust, simple genotyping-by-sequencing (GBS) approach for high diversity species. PLoS ONE.

[CR23] Esuma W, Herselman L, Labuschagne MT, Ramu P, Fei L, Baguma Y, Buckler ES, Kawuki RS (2016). Genome-wide association mapping of provitamin a carotenoid content in Cassava. Euphytica/Netherlands J Plant Breed.

[CR24] Fanou AA, Zinsou VA, Wydra K (2018). Cassava bacterial blight: a devastating disease of cassava. Cassava.

[CR25] Fan W, Hai M, Guo Y, Ding Z, Tie W, Ding X, Yan Y (2016). The ERF transcription factor family in Cassava: genome-wide characterization and expression analyses against drought stress. Sci Rep.

[CR26] Ferguson ME, Hearne SJ, Close TJ, Wanamaker S, Moskal WA, Town CD, de Young J, Marri PR, Rabbi IY, de Villiers EP (2012). Identification, validation and high-throughput genotyping of transcribed gene SNPs in Cassava. Theor Appl Genet.

[CR27] Ferguson ME, Shah T, Kulakow P, Ceballos H (2019). A global overview of cassava genetic diversity. PLoS ONE.

[CR28] Ferguson M, Rabbi I, Kim D-J, Gedil M, Lopez-Lavalle LAB, Okogbenin E (2012). Molecular markers and their application to cassava breeding: past, present and future. Tropical Plant Biology.

[CR29] Howeler R, Lutaladio N, and Thomas G. Food, and Agriculture Organization (2013) Save and grow: cassava: a guide to sustainable production intensification. Food and Agriculture Organization of the United Nations.

[CR30] Fregene M, Angel F, Gomez R, Rodriguez F, Chavarriaga P, Roca W, Tohme J, Bonierbale M (1997). A molecular genetic map of Cassava (*Manihot esculenta* Crantz). Theor Appl Genet.

[CR31] Gao L, Gonda I, Sun H, Ma Q, Bao K, Tieman DM, Burzynski-Chang EA (2019). The tomato pan-genome uncovers new genes and a rare allele regulating fruit flavor. Nat Genet.

[CR32] Garrison E, Sirén J, Novak AM, Hickey G, Eizenga JM, Dawson ET, Jones W (2018). Variation graph toolkit improves read mapping by representing genetic variation in the reference. Nat Biotechnol.

[CR33] Glaubitz JC, Casstevens TM, Fei L, Harriman J, Elshire RJ, Sun Q, Buckler ES (2014). TASSEL-GBS: a high capacity genotyping by sequencing analysis pipeline. PLoS ONE.

[CR34] Gomez MA, Daniel Lin Z, Moll T, Chauhan RD, Hayden L, Renninger K, Beyene G (2019). Simultaneous CRISPR/Cas9-mediated editing of cassava eIF4E isoforms nCBP-1 and nCBP-2 reduces cassava brown streak disease symptom severity and incidence. Plant Biotechnol J.

[CR35] Goodstein DM, Shu S, Howson R, Neupane R, Hayes RD, Fazo J, Mitros T (2012). Phytozome: a comparative platform for green plant genomics. Nucleic Acids Res.

[CR36] Gordon SP, Contreras-Moreira B, Woods DP, Des DL, Marais DB, Shu S, Stritt C (2017). Extensive gene content variation in the *Brachypodium distachyon* pan-genome correlates with population structure. Nat Commun.

[CR37] Guan D, McCarthy SA, Wood J, Howe K, Wang Y, Durbin R (2020). Identifying and removing haplotypic duplication in primary genome assemblies. Bioinformatics.

[CR38] Halsey ME, Olsen KM, Taylor NJ, Chavarriaga-Aguirre P (2008). Reproductive biology of cassava (*Manihot esculenta* Crantz) and isolation of experimental field trials. Crop Sci.

[CR39] Hamblin MT, Rabbi IY (2014). The effects of restriction-enzyme choice on properties of genotyping-by-sequencing libraries: a study in cassava (*Manihot esculenta*). Crop Sci.

[CR40] Hillocks RJ, Thresh JM, Bellotti A (2002). Cassava: biology, production and utilization.

[CR41] Hirsch CN, Foerster JM, Johnson JM, Sekhon RS, Muttoni G, Vaillancourt B, Peñagaricano F (2014). Insights into the maize pan-genome and pan-transcriptome. Plant Cell.

[CR42] Hummel AW, Chauhan RD, Cermak T, Mutka AM, Vijayaraghavan A, Boyher A, Starker CG, Bart R, Voytas DF, Taylor NJ (2018). Allele exchange at the EPSPS locus confers glyphosate tolerance in cassava. Plant Biotechnol J.

[CR43] Hu W, Hou X, Xia Z, Yan Y, Wei Y, Wang L, Zou M, Cheng L, Wang W, Peng M (2016). Genome-wide survey and expression analysis of the calcium-dependent protein kinase gene family in cassava. Mol Genet Genom: MGG.

[CR44] Hu W, Wei Y, Xia Z, Yan Y, Hou X, Zou M, Cheng L, Wang W, Peng M (2015). Genome-wide identification and expression analysis of the NAC Transcription factor family in cassava. PLoS ONE.

[CR45] Hu W, Yang H, Yan Y, Wei Y, Tie W, Ding Z, Zuo J, Peng M, Li K (2016). Genome-wide characterization and analysis of bZIP transcription factor gene family related to abiotic stress in cassava. Sci Rep.

[CR46] International Cassava Genetic Map Consortium (ICGMC) (2014). High-resolution linkage map and chromosome-scale genome assembly for cassava (*Manihot esculenta* Crantz) from 10 Populations. G3.

[CR47] International Rice Genome Sequencing Project (2005). The map-based sequence of the rice genome. Nature.

[CR48] Jennings DL (1976) Breeding for resistance to African cassava mosaic disease: progress and prospects. In: African cassava mosaic. IDRC, Ottawa, ON, CA

[CR49] Kayondo SI, Carpio DPD, Lozano R, Ozimati A, Wolfe M, Baguma Y, Gracen V (2018). Genome-wide association mapping and genomic prediction for CBSD resistance in *Manihot esculenta*. Sci Rep.

[CR50] Koren S, Rhie A, Walenz BP, Dilthey AT, Bickhart DM, Kingan SB, Hiendleder S, Williams JL, Smith TimothyPL, Phillippy AM (2018). De novo assembly of haplotype-resolved genomes with trio binning. Nat Biotechnol.

[CR51] Koren S, Walenz BP, Berlin K, Miller JR, Bergman NH, Phillippy AM (2017). Canu: scalable and accurate long-read assembly via adaptive K-Mer weighting and repeat separation. Genome Res.

[CR52] Kronenberg ZN, Hall RJ, Hiendleder S, Smith TimothyPL, Sullivan ST, Williams JL, Kingan SB (2018). FALCON-phase: integrating PacBio and Hi-C data for phased diploid genomes. bioRxiv.

[CR53] Kuon J-E, Qi W, Schläpfer P, Hirsch-Hoffmann M, Rogalla P, von Bieberstein A, Patrignani LP (2019). Haplotype-resolved genomes of geminivirus-resistant and geminivirus-susceptible African cassava cultivars. BMC Biol.

[CR54] Lander ES, Linton LM, Birren B, Nusbaum C, Zody MC, Baldwin J, Devon K (2001). Initial sequencing and analysis of the human genome. Nature.

[CR55] Léotard G, Duputié A, Kjellberg F, Douzery EmmanuelJP, Debain C, de Granville J-J, McKey D (2009). Phylogeography and the origin of cassava: new insights from the northern rim of the amazonian basin. Mol Phylogenet Evol.

[CR56] Liao W, Yang Y, Li Y, Wang G, Peng M (2016). Genome-wide identification of cassava R2R3 MYB family genes related to abscission zone separation after environmental-stress-induced abscission. Sci Rep.

[CR57] Lieberman-Aiden E, van Berkum NL, Williams L, Imakaev M, Ragoczy T, Telling A, Amit I (2009). Comprehensive mapping of long-range interactions reveals folding principles of the human genome. Science.

[CR58] Lin ZJD, Taylor NJ, Bart R (2019). Engineering disease-resistant cassava. Cold Spring Harb Perspect Biol.

[CR59] Li S, Xiang Yu, Lei N, Cheng Z, Zhao P, He Y, Wang W, Peng M (2017). Genome-wide identification and functional prediction of cold and/or drought-responsive lncRNAs in cassava. Sci Rep.

[CR60] Li Y-H, Zhou G, Ma J, Jiang W, Jin L-G, Zhang Z, Guo Y (2014). De novo assembly of soybean wild relatives for pan-genome analysis of diversity and agronomic traits. Nat Biotechnol.

[CR61] Michael TP, VanBuren R (2020). Building near-complete plant genomes. Curr Opin Plant Biol.

[CR62] Muñoz-Bodnar A, Perez-Quintero AL, Gomez-Cano F, Gil J, Michelmore R, Bernal A, Szurek B, Lopez C (2014). RNAseq analysis of cassava reveals similar plant responses upon infection with pathogenic and non-pathogenic strains of *Xanthomonas axonopodis* Pv. Manihotis. Plant Cell Rep.

[CR63] Nzuki I, Katari MS, Bredeson JV, Masumba E, Kapinga F, Salum K, Mkamilo GS (2017). QTL mapping for pest and disease resistance in cassava and coincidence of some QTL with introgression regions derived from *Manihot glaziovii*. Frontiers Plant Sci.

[CR64] Odipio J, Alicai T, Ingelbrecht I, Nusinow DA, Bart R, Taylor NJ (2017). Efficient CRISPR/Cas9 genome editing of phytoene desaturase in cassava. Frontiers Plant Sci.

[CR65] Olsen KM (2004). SNPs, SSRs and Inferences on Cassava’s ORIGIN. Plant Mol Biol.

[CR66] Olsen KM, Schaal BA (1999). Evidence on the origin of cassava: phylogeography of *Manihot esculenta*. Proc Natl Acad Sci USA.

[CR67] Olsen K, Schaal B (2001). Microsatellite variation in cassava (*Manihot esculenta*, Euphorbiaceae) and its wild relatives: further evidence for a Southern Amazonian origin of domestication. Am J Bot.

[CR68] Ou W, Mao X, Huang C, Tie W, Yan Y, Ding Z, Chunlai W (2018). Genome-wide identification and expression analysis of the KUP family under abiotic stress in cassava (*Manihot esculenta* Crantz). Frontiers Physiol.

[CR69] Padgitt-Cobb LK, Kingan SB, Wells J, Elser J, Kronmiller B, Moore D, Concepcion G (2019). A phased, diploid assembly of the cascade hop (*Humulus lupulus*) genome reveals patterns of selection and haplotype variation. Cold Spring Harbor Laboratory.

[CR70] Prochnik S, Marri PR, Desany B, Rabinowicz PD, Kodira C, Mohiuddin M, Rodriguez F (2012). The cassava genome: current progress, future directions. Tropic Plant Biol.

[CR71] Putnam NH, O’Connell BL, Stites JC, Rice BJ, Blanchette M, Calef R, Troll CJ (2016). chromosome-scale shotgun assembly using an in vitro method for long-range linkage. Genome Res.

[CR72] Rabbi I, Hamblin M, Gedil M, Kulakow P, Ferguson M, Ikpan AS, Ly D, Jannink J-L (2014). Genetic mapping using genotyping-by-sequencing in the clonally propagated cassava. Crop Sci.

[CR73] Rabbi IY, Hamblin MT, Lava Kumar P, Gedil MA, Ikpan AS, Jannink J-L, Kulakow PA (2014). High-resolution mapping of resistance to cassava mosaic geminiviruses in cassava using genotyping-by-sequencing and its implications for breeding. Virus Res.

[CR74] Rabbi IY, Udoh LI, Wolfe M, Parkes EY, Gedil MA, Dixon A, Ramu P, Jannink J-L, Kulakow P (2017). Genome-wide association mapping of correlated traits in cassava: dry matter and total carotenoid content. Plant Genome.

[CR75] Rabbi IY, Kulembeka HP, Masumba E, Marri PR, Ferguson M (2012). An EST-derived SNP and SSR genetic linkage map of cassava (*Manihot esculenta Crantz*). Theor Appl Genet.

[CR76] Ramu P, Esuma W, Kawuki R, Rabbi IY, Egesi C, Bredeson JV, Bart RS, Verma J, Buckler ES, Fei L (2017). Cassava haplotype map highlights fixation of deleterious mutations during clonal propagation. Nat Genet.

[CR77] Rauwane ME, Odeny DA, Millar I, Rey C, Rees J (2018). The early transcriptome response of cassava (*Manihot esculenta* Crantz) to mealybug (*Phenacoccus manihoti*) feeding. PLoS ONE.

[CR78] Roach MJ, Schmidt SA, Borneman AR (2018). Purge haplotigs: allelic contig reassignment for third-gen diploid genome assemblies. BMC Bioinf.

[CR79] Soto JC, Ortiz JF, Perlaza-Jiménez L, Vásquez AX, Lopez-Lavalle LAB, Mathew B, Léon J, Bernal AJ, Ballvora A, López CE (2015). A genetic map of cassava (*Manihot esculenta* Crantz) with integrated physical mapping of immunity-related genes. BMC Genom.

[CR80] Tecle IY, Edwards JD, Menda N, Egesi C, Rabbi IY, Kulakow P, Kawuki R, Jannink J-L, Mueller LA (2014). solGS: a web-based tool for genomic selection. BMC Bioinf.

[CR81] The NCBI Handbook (2013) National Center for Biotechnology Information (US)

[CR82] Vanderschuren H, Nyaboga E, Poon JS, Baerenfaller K, Grossmann J, Hirsch-Hoffmann M, Kirchgessner N, Nanni P, Gruissem W (2014). Large-scale proteomics of the cassava storage root and identification of a target gene to reduce postharvest deterioration. Plant Cell.

[CR83] Wang H, Beyene G, Zhai J, Feng S, Fahlgren N, Taylor NJ, Bart R, Carrington JC, Jacobsen SE, Ausin I (2015). CG gene body DNA methylation changes and evolution of duplicated genes in cassava. Proc Natl Acad Sci USA.

[CR84] Wang W, Feng B, Xiao J, Xia Z, Zhou X, Li P, Zhang W (2014). Cassava genome from a wild ancestor to cultivated varieties. Nat Commun.

[CR85] Wang X, Chang L, Tong Z, Wang D, Yin Q, Wang D, Jin X (2016). Proteomics profiling reveals carbohydrate metabolic enzymes and 14-3-3 proteins play important roles for starch accumulation during cassava root tuberization. Sci Rep.

[CR86] Wei Y, Shi H, Xia Z, Tie W, Ding Z, Yan Y, Wang W, Wei H, Li K (2016). Genome-wide identification and expression analysis of the WRKY gene family in cassava. Frontiers Plant Sci.

[CR87] Wilson MC, Mutka AM, Hummel AW, Berry J, Chauhan RD, Vijayaraghavan A, Taylor NJ, Voytas DF, Chitwood DH, Bart RS (2017). Gene expression atlas for the food security crop cassava. New Phytol.

[CR88] Wolfe MD, Bauchet GJ, Chan AW, Lozano R, Ramu P, Egesi C, Kawuk R, Kulakow P, Rabbi I, Jannink J-L (2019). Historical introgressions from a wild relative of modern cassava improved important traits and may be under balancing selection. Genetics.

[CR89] Wolfe MD, Kulakow P, Rabbi IY, Jannink J-L (2016). Marker-based estimates reveal significant nonadditive effects in clonally propagated Cassava (*Manihot esculenta*): implications for the prediction of total genetic value and the selection of varieties. G3.

[CR90] Wolfe MD, Rabbi IY, Egesi C, Hamblin M, Kawuki R, Kulakow P, Lozano R, Del Carpio DP, Ramu P, Jannink J-L (2016). Genome-wide association and prediction reveals genetic architecture of cassava mosaic disease resistance and prospects for rapid genetic improvement. Plant Genome.

[CR91] Zhang Y, Ding Z, Ma F, Chauhan RD, Allen DK, Brutnell TP, Wang W, Peng M, Li P (2015). Transcriptional response to petiole heat girdling in cassava. Sci Rep.

[CR92] Zhao Q, Feng Q, Hengyun L, Li Y, Wang A, Tian Q, Zhan Q (2018). Pan-genome analysis highlights the extent of genomic variation in cultivated and wild rice. Nat Genet.

[CR93] Zou M, Cheng L, Zhang S, Chen Q, Sun X, Ma P, Meizhen H (2017). Epigenetic map and genetic map basis of complex traits in cassava population. Sci Rep.

