# Correction to: Current status and impending progress for cassava structural genomics

**DOI:** 10.1007/s11103-021-01139-7

**Published:** 2021-04-13

**Authors:** Jessica B. Lyons, Jessen V. Bredeson, Ben N. Mansfeld, Guillaume Jean Bauchet, Jeffrey Berry, Adam Boyher, Lukas A. Mueller, Daniel S. Rokhsar, Rebecca S. Bart

**Affiliations:** 1 Department of Molecular & Cell Biology, University of California, Berkeley, CA 94720 USA; 2 Innovative Genomics Institute, University of California, Berkeley, CA 94720 USA; 3 Donald Danforth Plant Science Center (DDPSC), St. Louis, MO 63132 USA; 4 Boyce Thompson Institute, Ithaca, NY 14853 USA; 5 DOE Joint Genome Institute, Walnut Creek, CA USA; 6 Chan-Zuckerberg BioHub, 499 Illinois, San Francisco, CA 94158 USA

## Correction to: Plant Molecular Biology 10.1007/s11103-020-01104-w

In the above mentioned publication, some of the authors' affiliations weren't correctly identified. The original article has been corrected and the proper representation of the authors and their affiliations is also published here.

